# Photo-Controlled Self-Assembly of Nanoparticles: A Promising Strategy for Development of Novel Structures

**DOI:** 10.3390/nano13182562

**Published:** 2023-09-15

**Authors:** Juntan Li, Xiaoyong Jia

**Affiliations:** Henan Key Laboratory of Photovoltaic Materials, College of Future Technical, Henan University, Kaifeng 475004, China; lijt1996@163.com

**Keywords:** photo controlled, nanoparticles, self-assembly, photoresponsive molecules, photoexcitation, excited state

## Abstract

Photo-controlled self-assembly of nanoparticles (NPs) is an advanced and promising approach to address a series of material issues from the molecular level to the nano/micro scale, owing to the fact that light stimulus is typically precise and rapid, and can provide contactless spatial and temporal control. The traditional photo-controlled assembly of NPs is based on photochemical processes through NPs modified by photo-responsive molecules, which are realized through the change in chemical structure under irradiation. Moreover, photoexcitation-induced assembly of NPs is another promising physical strategy, and such a strategy aims to employ molecular conformational change in the excited state (rather than the chemical structure) to drive molecular motion and assembly. The exploration and control of NP assembly through such a photo-controlled strategy can open a new paradigm for scientists to deal with “bottom-up” behaviors and develop unprecedented optoelectronic functional materials.

## 1. Introduction

Molecular self-assembly is the most common phenomenon in nature, where small molecules spontaneously combine to form large, ordered structures [1,2]. Through molecular self-assembly, ordered aggregates with sizes ranging from nanometers to micrometers can be obtained, such as protein, colloids, nanoparticles (NPs), and double helical DNA [3,4,5]. NP self-assembly has emerged as one of the most important concepts in nanoscience and nanotechnology, which are widely regarded as the mainstream technologies of the 21st century, focusing on observing and manipulating substances at the nanoscale [6,7,8]. Although NP self-assembly has been extensively studied, achieving precise regulation of molecular self-assembly through effective means remains challenging. Researchers have made tremendous efforts to conveniently construct assembled NPs with novel structures and functions [9,10,11,12]. Therefore, significant research achievements and progress have been made in exploring the nanoworld.

A promising strategy for exploring the self-assembly of NPs is to introduce external stimuli to regulate the self-assembly environment or molecular structure [13,14,15]. In this regard, numerous self-assembled NPs that can respond to various external stimuli, such as temperature, light, pH, solvent polarity, electrical currents, magnetism, and ions, have been developed [16,17,18,19,20,21]. Among these stimulus-responsive nanosystems, light has been proven to be a unique and effective regulation approach because of its unique, clean, and non-invasive properties. Light has obvious advantages as a means of regulation. First, the intensity and wavelength of light can be flexibly adjusted according to the system characteristics. Second, light can be transmitted to the target location remotely in a non-contact manner, which can be easily and quickly used and removed [22,23,24,25]. Therefore, in the field of self-assembly technology, photo-driven NP self-assembly has become a smart and key strategy for adjusting assembled structures and functions, which can effectively achieve precise control of the assembled nanostructures and properties, and has attracted increasing attention from researchers in recent years [26,27,28,29,30].

In the past few years, two main methods of photo-controlled self-assembly with reversible behavior have been developed: self-assembly based on photochemical processes and self-assembly based on photophysical processes [31]. The most studied and widely used method is the introduction of photoresponsive molecules into nanosystems based on photochemical processes, which can endow NPs with photoresponsive properties [32]. Such photochemical process-induced self-assembly is typically achieved through photoreaction, photocyclization, and photoisomerization, including chemical structural changes to photochromic molecules in single or multi-component material systems. Generally, changes in the molecular structure before and after photochemical reactions induce the self-assembly process of the entire molecular system in two states. The other type of photo-controlled self-assembly is achieved based on photophysical processes [31]. Compared with photochemical processes, photophysical processes have unique advantages. Photophysical processes do not require the presence of photoresponsive groups or structural changes in molecules. Therefore, photophysical processes may apply to most molecular systems as such molecules are easier to design and synthesize, more environmentally friendly, and easier to control.

Previous reviews are mostly focused on the self-assembly of NPs controlled by the photochemical process and, therefore, the self-assembly of NPs controlled by the photophysical process is rare. Moreover, there has been no review on the self-assembly of NPs controlled by photochemical and photophysical processes. Therefore, in this review, we summarize the two together to better interpret the concept of photo-controlled NP self-assembly in recent years. First, we introduce the photochemical process-controlled self-assembly of NPs modified through photochromic systems and illustrate the development of NP assemblies from such photoresponsive compounds. Second, we discuss the photophysical process and the photo-controlled self-assembly of NPs modified through polyphenylthiobenzene based on excited state conformational changes. Then, we provide a summary and outlook for further development of photo-controlled NP self-assembly.

## 2. Photochemical Process-Controlled Self-Assembly of NPs Based on Photochromic Molecules

### 2.1. Introduction to Photochemical Process

**Figure 1:**
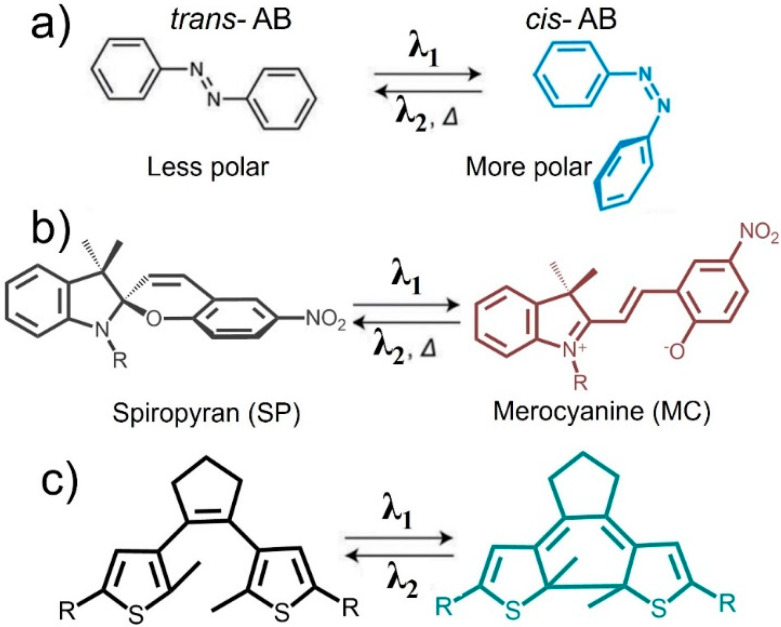
The most commonly used photoswitches based on the photochemical process. (**a**) Azobenzene, (**b**) spiropyran, (**c**) dithienylethene.

Photochemical process-controlled self-assembly is achieved by combining photochromic molecules with biomacromolecules, NPs, polymers, etc., through covalent or non-covalent interactions, using the structural changes to photochromic molecules as the driving force [23,32]. Up to now, the extensively studied photochromic molecular systems mainly include azobenzenes, spiropyrans, and diarylethylenes (Figure 1) [33,34,35]. These systems use the formation and breaking of chemical bonds under light to form new molecular structures through photochemical reactions, such as isomerization, addition, open/closed ring, and redox, thereby driving the self-assembly process of the entire system. Three main, effective, photoswitch self-assembly systems are introduced in this review, due to the wider research scope, potential applications, and more effective design ideas associated with these systems. During the reversible isomerization of these photoswitches, their properties, including polarity, appearance, luminescence, or bondability with ions, typically change, resulting in differences in the functionality and structure of the assembly.

### 2.2. Photo-Controlled Self-Assembly of Nanoparticles

NPs have been widely studied in various scientific fields because of their unique properties, which can be tuned by controlling the size and surface chemistry, leading to their widespread application [36,37]. The directional regulation of the self-assembly of NPs is a fundamental challenge and has significant implications for developing the next generation of advanced structural and functional materials. Functionalized NPs, using the aforementioned photoresponsive molecules, will endow NPs with photoresponsibility, thereby realizing self-assembly controlled through light. Although the use of photoresponsive molecular functionalization to modify NPs has achieved significant success, this method is not the only way to obtain photoresponsive NPs. In contrast, in recent years, various creative methods have been developed that utilize light to control NP self-assembly. These methods include photo-induced reversible covalent bond formation, protonation and deprotonation of NP-bound ligands using small molecular photoacids and photobases, photo-controlled deposition and desorption of optical switch molecules on NPs, phase transition of thermal responsive polymers bound to NPs induced by plasma NPs, and photo-induced electron transfer between the NP core and ligand [38,39].

#### 2.2.1. Azobenzene-Functionalized Nanoparticles

Azobenzene (azo) is an extensively studied photoresponsive molecule that can undergo cis-trans photoisomerization with fast switching (Figure 1a). Trans-azobenzene is a planar molecule with zero dipole moment that can switch to the cis isomer that is nonplanar and has a dipole moment of 3 D after ultraviolet (UV) irradiation. Meanwhile, cis-azobenzene can return to a trans-isomer with visible light (460 nm) irradiation. This performance can be regulated through changes in the core substituents of azobenzene and the selection of solvents [40,41]. It is the structural changes before and after isomerization that provide the basis for developing photoresponsive self-assembly systems using azobenzene.

Azobenzene is highly suitable for inducing the reversible self-assembly of NPs because of the following reasons. First, its derivatives can be easily synthesized and have good compatibility with various surface functional groups of inorganic NPs, providing a foundation for binding with NPs. Second, changing the substituents on the azobenzene core can significantly impact the photoisomerization wavelength, isomerization molecular structure, and dipole moment. In particular, significant changes in the molecular structure (from the *trans*-planar and elongated structure to the *cis* three-dimensional structure) play a key role in the process of photo-controlled self-assembly [42]. At present, azobenzene derivatives are widely used in the development of photo-controlled NP self-assembly, biomolecular self-assembly, and polymer phase transition [43,44].

The self-assembly of noble metal NPs, such as gold (Au) and silver (Ag) NPs, has been widely explored because of the astonishing properties of these NPs, especially their size-dependent properties, which can be achieved through the assembly or aggregation of NPs [45,46,47]. Tuning the self-assembly process of noble metal NPs using the photoresponsivity of azobenzene molecules is simple and effective.

**Figure 2:**
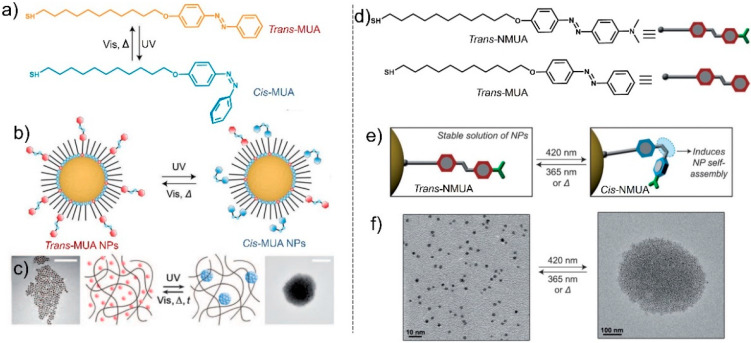
(**a**) *Trans*–*cis* isomerism structural formula for 4-(11-mercaptoundecanoxy)azobenzene. (**b**) Schematic illustration of the photoisomerization process of gold nanoparticles modified with MUA and dodecylamine (DDA). (**c**) Photo-controlled aggregation of NPs through photoisomerization [48]. (**d**) Structural formulas of the thiolated azobenzenes (*trans*-NMUA). (**e**) Reversible photoisomerization of *trans*-NMUA on the surface of the NPs. (**f**) TEM images of the photo-controlled assembly (**right**) and disassembly (**left**) of the Au NPs functionalized by *trans*-NMUA [49].

Grzybowski et al. demonstrated the photo-controlled dynamic non-equilibrium, reversible self-assembly of Au NPs modified with an azobenzene derivative named *trans*-MUA (Figure 2a) [48]. In the initial state, MUA on the surface of the NPs exists in the *trans* form with a small dipole moment. In this case, the attractive interactions between the NPs were weak, resulting in the inability of the NPs to effectively self-assemble and exhibit dispersed morphology (Figure 2b). Under UV irradiation, the initial *trans*-MUA can switch to *cis*-MUA, accompanied by a significant increase in the dipole moment (from 1 D to 5 D). Thus, the attractive interaction between the NPs is strengthened, and the NPs can self-assemble to form aggregated NPs (Figure 2c). Once the *cis*-MUA returns to the trans-MUA, the aggregated NPs can recover to the pristine state, exhibiting good reversibility.

Nevertheless, it is more meaningful to obtain NPs with visible light-controlled self-assembly. Many studies have shown that through an effective substituent modification, the absorption wavelength of photoresponsive molecules can be regulated from the UV region to the visible light region. Klajn et al. prepared Au NPs and modified the surface with different substituted azobenzenes (MUA and NMUA, Figure 2d), which can respond to visible light [49]. By introducing dimethylamino into the para position of MUA, the dipole moment of NMUA can significantly increase, with the *trans* formula being 4.65 D and the *cis* formula being 6.7 D, indicating that NPs decorated with NMUA can undergo self-assembly in nonpolar solvents. Meanwhile, the introduction of dimethylamino groups enables molecules to exhibit absorption in the visible light range. Therefore, NPs decorated with NMUA can exhibit visible light-controlled self-assembly (Figure 2f). The obtained NPs decorated by *trans*-NMUA can form 2.5 nm Au NPs, whereas the trans-NMUA can switch to *cis*-NMUA under 420 nm irradiation, further triggering the self-assembly of aggregates of the 2.5 nm Au NPs to form larger aggregated Au NPs (Figure 2f). Interestingly, when NPs are decorated with NMUA and MUA, the driving force of the self-assembly for NPs can be driven by the isomerization of NMUA and MUA, respectively. Under 365 nm irradiation, *cis*-NMUA can transform to *trans*-NMUA and *trans*-MUA can transform to *cis*-MUA; thus, the trans–cis isomerization of MUA triggered the assembly of NPs. Meanwhile, under 420 nm irradiation, *trans*-NMUA can transform to *cis*-NMUA and trans-MUA exhibits no response; thus, the trans–cis isomerization of NMUA triggered the assembly of NPs. Therefore, modifying NPs using multi-component compounds with multi-stimuli-responsive properties is expected to be a new approach for developing novel nanostructures and multifunctional materials.

**Figure 3:**
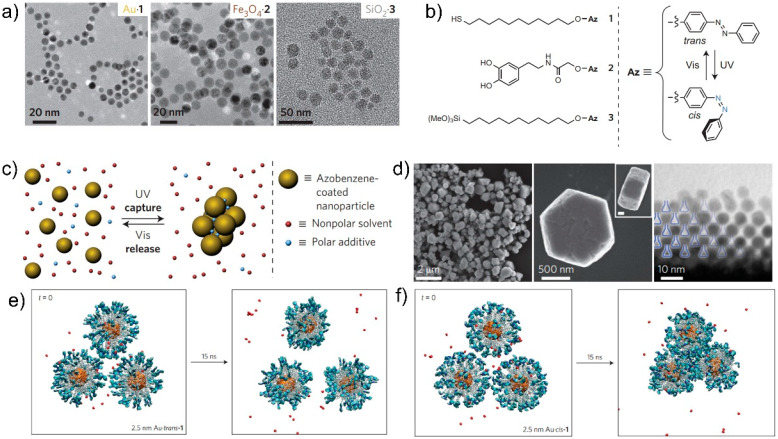
(**a**) TEM images of photoresponsive Au, Fe_3_O_4_, and SiO_2_ nanoparticles (**left** to **right**) used to generate dynamically self-assembling nanoflasks. (**b**) Structural formulae of ligands 1–3 used to modify the NPs. (**c**) Schematic illustration of the reversible trapping of polar molecules through photo-controlled self-assembly of NPs. Photo-controlled aggregation of NPs through photoisomerization. (**d**) Electron micrographs (at different magnifications) of colloidal crystals prepared through UV irradiation of Au NPs modified with 1. (**e**) Snapshots from atomistic simulations of *trans*-1-functionalized and (**f**) *cis*-1-functionalized Au NPs in toluene saturated with water [51].

NP self-assembly based on photo-induced cis–trans isomerization of azobenzene has been used to construct well-ordered three-dimensional superstructures, such as crystals and supraspheres of various sizes with optically reversible or irreversible behavior. Such reversible performance can be determined by the dipole–dipole interaction strength between the NPs and covalent binding of the NPs. Temps et al. obtained a series of azobenzene and alkyl thiol modified Au NPs by changing the length of the alkyl chain. They found that the short-chain azobenzene in the long-chain thiol matrix can effectively undergo photoisomerization, but does not promote NP self-assembly [50]. In addition, the concentration of dithiol ligands can have a significant impact on the assembly of NPs. The assembled three-dimensional crystals have high reversibility and can switch between disordered and crystalline states when the surface concentration of dithiol ligands is low. In contrast, the NP assemblies can maintain high stability because they can be cross-linked because of higher concentrations of dithiol ligands [51]. Klajn et al. proved that by decorating Au NPs with azobenzenes, photo-controlled directional dynamic self-assembly to form nanoflasks can be achieved (Figure 3). As shown in Figure 3b, the dipole moment of azobenzene increased after trans-to-cis isomerization under UV irradiation, and attractive dipole–diploe interactions can form among the functionalized NPs. Once the dipole–dipole interaction force can overcome the repulsions among the NPs, the NPs self-assemble to form nanoflasks (Figure 3d). During the self-assembly process, various molecules in the surrounding bulk solution can be captured (Figure 3c), and under visible light irradiation, nanoflasks can de-assemble to release the captured molecules back into the bulk solution, only in the case of *cis*-azobenzene nanoparticle aggregation, and the trapping of water is observed (Figure 3e,f). These studies provide a new approach for the direct photo-controlled synthesis of nanostructures and multifunctional devices.

**Figure 4:**
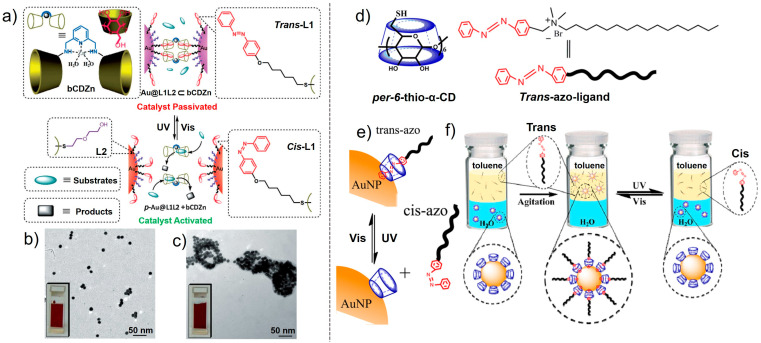
(**a**) Structures of the supramolecular catalyst bCDZn, the ligand components, L1 and L2, and the proposed mechanism and schematic representation of the phototriggered interparticle host–guest competitive binding for switchable catalysis. (**b**) TEM images of Au@L1L2 and (**c**) after the addition of 20 equivalent bCDZn through the phototriggered assembly/disassembly process in water at 298 K [52]. (**d**) Structures of the host per-6-thio-α-CD and guest *trans*-azo ligand. (**e**) Photoreversible inclusion of the azo ligand in α-CD-coated Au NPs. (**f**) Photo-controlled phase transfer of α-CD-capped Au NPs by azo ligands between water and toluene [53].

Regarding azobenzene ligand-modified NPs, the introduction of host–guest interactions is also an effective way to achieve photo-controlled self-assembly [52]. Zhao et al. designed a host–guest supramolecular catalyst consisting of a Zn-coordinated β-CD dimer host and an Au NP guest decorated by azobenzene (Figure 4a). In the initial state, the β-CD rings are occupied by the trans-azobenzene unit and catalytic activity is passivated because the supramolecular catalyst cannot be recognized by the reaction substrate (Figure 4b). Once the trans-azobenzene transforms to cis-azobenzene after UV irradiation, cis-azobenzene cannot bind well with β-CD, thereby activating the catalyst activity to achieve a hydrolysis reaction of the catalytic substrate (Figure 4c). Meanwhile, *cis*-azobenzene can recover to trans-azobenzene under visible light irradiation, terminating the catalytic process. Such a catalytic process can be switched on/off by controlling the trans–cis isomerization of azobenzene, which provides a foundation for exploring next-generation photoresponsive catalytic materials. By utilizing the interaction between the host of the Au NPs coated with α-CD and the guest of azobenzene, Tan et al. achieved photo-controlled reversible phase transfer between toluene and an aqueous phase [53]. A *trans*-azo ligand with hydrophobic alkyl chains that can effectively dissolve in toluene and Au NPs with good water solubility coated with α-CD were designed (Figure 4d). Initially, the *trans*-azo ligand can bind well with α-CDs due to the host–guest interaction, resulting in the formation of a reverse micelle-like structure because of the presence of hydrophobic alkyl chains and the conversion of the Au NPs from hydrophilic to hydrophobic, thereby transferring from water to toluene. (Figure 4f) In contrast, when the *trans*-azo switches to a *cis*-azo ligand after UV irradiation (Figure 4e), a host–guest interaction cannot be effectively formed between *cis*-azo and α-CDs, leading to the dissociation of *cis*-azo from the Au NPs. In this case, the hydrophilic property of Au NPs will recover, thereby promoting the transfer of Au NPs from toluene to water (Figure 4f). As a result, photo-controlled reversible toluene–water phase transfer was achieved, paving the way for the development of photo-controlled functional hydrophilic and hydrophobic transition materials.

In addition, using azobenzene to modify magnetic NPs, biomacromolecules, and chiral molecules to realize optically controlled self-assembly is an effective and feasible approach [54,55,56,57,58,59].

#### 2.2.2. Spiropyran-Functionalized Nanoparticles

Spiropyran derivatives are another type of molecular switch that can transition from nonpolar to polar metastable states under light induction and can undergo photochemical cleavage of the C-O bond under UV irradiation, resulting in the transformation of the initial colorless state into a colored zwitterionic planar merocyanine form, which can promote the self-assembly of NPs in nonpolar media. The closed-ring spiropyran (SP in Figure 1b) transforms into the corresponding open-ring isomer (“subcyanidin”; MC) after UV irradiation. The polar MC isomer can recover to SP in the dark because it is unstable in nonpolar solvents. Meanwhile, the MC isomer can recover to SP under visible light. This characteristic provides the possibility to achieve photo-controlled self-assembly of NPs modified by SP. Light-controlled self-assemblies of supramolecules or NPs with unique functions have been developed utilizing such significant isomerization of these molecules [60,61,62,63].

**Figure 5:**
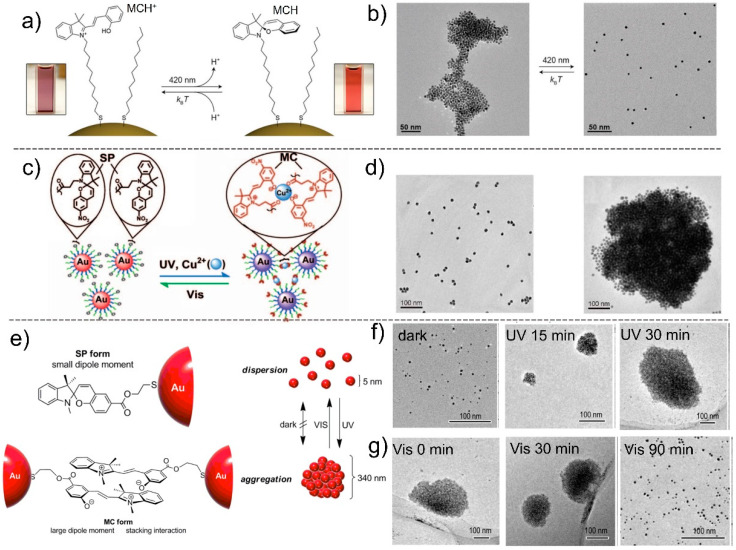
(**a**) Switch between MCH^+^ and SP-coated Au NPs under light in toluene. (**b**) TEM images of assembly/disassembly of NPs controlled by light [64]. (**c**) Proposed mechanism for the aggregation of Au NPs through photo-controlled formation/dissociation of Cu-coordination bond. (**d**) TEM images of photo-controlled assembly/disassembly of Au NPs through coordination bond formation/dissociation. Structures of host per-6-thio-α-CD and guest *trans*-azo ligand [65]. (**e**) Mechanism for phototriggered reversible aggregation/dispersion of AuNPs modified with SP. (**f**) TEM images of photo-controlled aggregation of SP–Au NPs and (**g**) dispersion of aggregated SP–Au NPs in toluene upon UV irradiation with time increasing [66].

Klajn et al. used an SP photoswitch to modify the surface of Au and Ag NPs, obtaining a novel type of photoresponsive NPs [64]. The SP-decorated NPs can self-assemble to form spherical aggregates in nonpolar solvents under UV irradiation, which is caused by the transformation of nonpolar SP into polar MC form (Figure 5a). The aggregated NPs can disassemble quickly following the removal of UV irradiation, due to the recovery of the SP form. By introducing other long alkyl chains, the assembly behavior can be tuned. The concentration of NPs can determine the size of aggregates, and the surface concentration of SP on NPs can control the life of dynamic NP aggregates (Figure 5b). Notably, the assembled NPs are easily disassembled when exposed to blue light.

When the SP form isomerized to the MC form after UV irradiation, a phenolate group is generated, which can easily combine with metal ions to form a coordination bond. Such coordination bonds can be dissociated under visible light due to the return of the MC form to the SP form [65]. Jiang et al. proposed a new strategy for realizing photo-controlled self-assembly based on SP–Au NP and Cu^2+^ complex systems (Figure 5c). The pristine Au NPs modified by alkanethiol terminated in SP and alkanethiol terminated in triethylene glycol exist in a dispersed state, and in this case, Cu^2+^ cannot form a coordination bond with SP. After UV irradiation, the SP form returns to the MC form that can coordinate with Cu^2+^, resulting in the formation of cross-linked structures and aggregated NPs through coordination bonds (Figure 5d). Other metal ions can also cause the aggregation of NPs, but certain concentration conditions need to be met. Therefore, the MC form exhibits selectivity for Cu^2+^ at low concentrations. This can be attributed to the higher water exchange rate constant of Cu^2+^ than other ions, indicating that kinetically, Cu^2+^ has more opportunities to combine with MC isomers. This phenomenon can be well used in the application of photo-controlled reversible aggregation in logic gates.

After transitioning from the SP form to the MC form, the dipole moment underwent significant changes, from a small dipole moment to a large dipole moment with a charge separation form. Therefore, the electrostatic repulsion/attraction may be controlled through this strategy [66]. Hirai et al. proved the feasibility of this strategy in photo-controlled aggregates of Au NPs modified by thiol-terminated spiropyran (Figure 5e). The initial NPs can form particles with a 5 nm diameter, whereas with an increase in the UV irradiation time, larger NP aggregates can form gradually, and aggregates with a 330 nm diameter are formed after 30 min irradiation (Figure 5f). Meanwhile, the aggregates with a 330 nm diameter can gradually dissemble to form 5 nm particles under 90 min visible light irradiation (Figure 5g). The MC isomer formed here has a large dipole moment under UV irradiation, which can promote the stacking interaction and association with each other. This phenomenon not only suppresses the recovery of MC to SP, but also maintains the electrostatic attraction between Au NPs. Therefore, the disassembly process of aggregated NPs requires visible light irradiation. The strategy proposed here may help in designing a more effective photo-induced self-assembly method for NPs and open up a new approach for the advanced processing of metal NPs.

**Figure 6:**
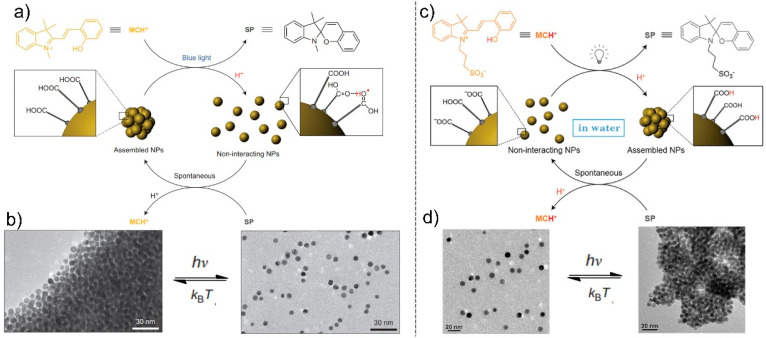
(**a**) Photoswitchable between MCH^+^ and SP-coated Au NPs that controlled the assembly state of NPs in organic solvent. (**b**) TEM images of NPs that switch between dispersed (**left**) and aggregated (**right**) state controlled by light [26]. (**c**) Photo-controlled assembly of MHA-coated NPs in water. (**d**) TEM images of dispersed (**left**) and aggregated (**right**) state of NPs controlled by light in water [67].

The transformation of spiropyran from the SP form to the MC form can be controlled by light, which can realize the formation and dissociation of coordination bonds and control the formation and dissociation of hydrogen bonds through photo-induced proton release or capture, thereby controlling the self-assembly process. Klajn et al. used photoacid responsive NPs in a “photoresponsive medium” (a dilute solution of spiropyran in methanol) to achieve NP assembly through the formation and dissociation of hydrogen bonds under photo stimuli [26]. The MC isomer of spiropyran can transform to a protonated MCH^+^ form under acidic conditions. Meanwhile, the MCH^+^ form can return to the SP form and release protons under visible light (Figure 6a). This transition provides the possibility to achieve photo-controlled hydrogen bonding self-assembly. Au NPs modified by 11-mercaptoundecanoic acid (MUA) can aggregate in various solvents due to interparticle interactions, because of multiple hydrogen bonds of COOH groups. Such hydrogen bonds are broken in the presence of acid, and the aggregated NPs disassemble to form individual particles. When NPs modified by MUA combine with MCH^+^, they transform to the SP form and release H^+^ ions under visible light. The released H^+^ ions can combine with COOH groups at the NP surface to form hydrogen bonds. Therefore, the assembled NPs disassemble to form individual particles (Figure 6b). Meanwhile, the SP form can recapture the H^+^ ions when the excitation source is removed, causing the re-assembly of individual NPs. This strategy proves that introducing photoresponsive groups into non-photoresponsive NP systems can effectively improve the photoresponsivity of NPs, providing a new direction for developing more photo-controlled self-assembly systems. This strategy is used only in organic solvents; however, controlling the self-assembly of NPs in aqueous solutions remains of great significance.

To achieve photo-controlled self-assembly of NPs in aqueous solutions, Klajn et al. modified NPs with 6-mercaptohexanoic acid (MHA) by replacing the MUA (Figure 6c) [67]. The shorter length of MHA promotes attractive interactions between NPs. Through the addition of tetramethylammonium hydroxide (TMA^+^OH^−^), COOH groups can deprotonate to form the COO^−^ group, making the interaction between NPs disappear and dissolve in water. Therefore, the MCH^+^ and NP systems can disperse well in aqueous solutions. Under visible light irradiation, MCH^+^ can transform into the SP form and release H^+^ ions. The released H^+^ ions can combine with the COO^−^ group to reform the COOH group, causing the enhancement of the interaction between NPs. Accordingly, NPs re-assemble to form aggregated NPs. Once the NP aggregates lie in the dark, SP transforms into MCH^+^ by capturing the protons of the COOH group, accompanied by the disassembly of NPs (Figure 6d).

#### 2.2.3. Diarylethene-Functionalized Nanoparticles

Diarylethene (DAE) derivatives can undergo reversible transformation between a colorless ring-open form and a colored ring-closed form induced by light, and have received increasing research interest because they are highly fatigue resistant and have excellent thermal stability (Figure 1c). Typically, the side cis–trans isomerization of styrene is suppressed by the introduction of a small ring skeleton. Thus far, research interest has mainly focused on the modification of side aryl rings [68]. Therefore, the absorption wavelengths can be modified to the visible light region using substituents, such as the extension of a conjugated system or the connection of a triplet sensitizer. Up to now, chiral molecules, nucleosides, and metallacycles modified with DAEs have been proven to be effective and promising in fabricating photo-controlled self-assembly systems [69]. There are significant changes in the photophysical properties before and after photoisomerization. Numerous efforts have been made to study the isomerization mechanism of DAEs. The switch reversibility and fatigue resistance can be improved through the substitution pattern of ethene bridges and aryl groups. Due to the significant structural differences between isomers, DAE molecules undergo self-assembly behavior in aggregates or solids, resulting in ordered nanostructures. Compared with other photoswitch molecules, research on the photo-controlled self-assembly of NPs modified by DAEs is rare. As a result, there is a little introduction in the following content. However, DAEs are widely used for surface functional modification of NPs [70,71], iron complexes [72], and host–guest self-assembly [73,74,75,76].

## 3. Photophysical Process-Controlled Self-Assembly of NPs

### 3.1. Photophysical Process

Photophysical processes are environmentally friendly and easy to control, and have attracted extensive research attention in recent years. In particular, photophysical processes based on spatial conformational changes to excited states have unique advantages. First, a molecule can quickly change from the ground to excited states under light excitation, and this change can be quickly recovered when the excitation source is removed, providing a basis for realizing real-time and in situ regulation. Second, changes in the spatial conformation between the ground and excited states of the molecule belong to a photophysical process, and the molecular structure remains unchanged. This prevents the formation of by-products and the problem of incomplete reaction conversion, which can effectively reduce the existence of assembly defects. Finally, the entire process is based on the excited state conformational change, which does not require the introduction of traditional photoresponsive molecules; thus, it is more conducive to the development of molecular systems. More attention has been paid to the regulation of molecular structures and properties in the excited state, and some material systems based on conformational changes in the excited state have been developed [77,78,79,80]. Up to now, photo-controlled crystal growth, photo-regulated luminescence, and photo-controlled aggregation have been realized in multiple molecular systems by tuning the excited state conformation [81,82,83]. Among these, the most systematic research has been conducted on the photo-controlled behavior of polyphenylthiobenzene molecules.

### 3.2. Photo-Controlled Behavior Polyphenylthiobenzene Derivatives

Previous studies have demonstrated that polyphenylthiobenzene derivatives have rich and easily controlled optical properties, as well as molecular crystals under different self-assembly driving forces. Therefore, these compounds have various external stimuli (light, pH, ion, force, and solvent)-responsive behavior, exhibiting reversible changes in optical properties and molecular configurations under stimuli [84,85,86,87,88,89]. Polyphenylthiobenzene derivatives are a type of “star-shaped” twisted molecules with flexible and rotatable C–S–C bonds, which is conducive of the obvious difference between their molecular excited and ground state conformations. The multiple non-covalent interactions (including C–H … π, π … π, S–S, and hydrogen halide bonds) formed between molecules can further drive self-assembly. Moreover, the heavy-atom effect of polysulfide endows these molecules with a long-lived excited state, which guarantees the aggregation and self-assembly of excited molecules through motion and collision on a time scale. Based on these characteristics of polyphenylthiobenzene molecules, photo-controlled molecular aggregation in solution, photo-controlled crystal growth and realignment, photo-induced block copolymer directed self-assembly in organic phases, photo-regulated microphase separation–recognized circularly polarized luminescence, and photo-tuning phase transformation have been achieved [90,91,92].

#### 3.2.1. Photo-Controlled Molecular Aggregation

**Figure 7:**
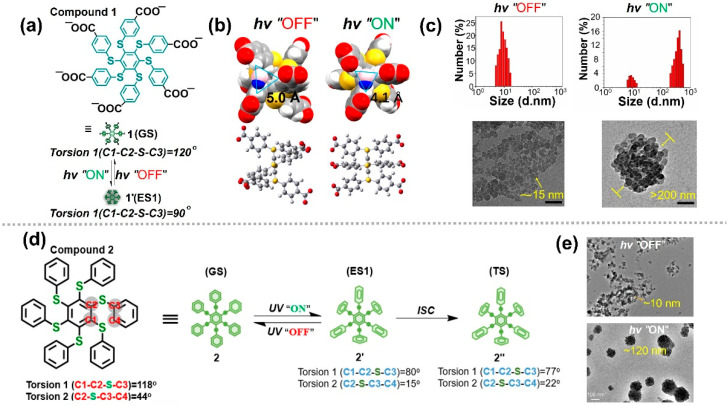
(**a**) Schematic illustration of the proposed conformational change upon photoexcitation of compound **1**. (**b**) Equilibrium geometry of the GS and ES1 state of compound **1**. (**c**) DLS results (**top**) and TEM images (**bottom**) of compound **1** in water before and after irradiation for 5 min [93]. (**d**) Calculated conformational change upon photoexcitation of compound **2**. (**e**) TEM images of compound **2** in organic solvent before and after irradiation [94].

Zhu et al. designed a polyphenylthiobenzene molecule (compound **1**, Figure 7a) that can effectively dissolve in aqueous solutions [93]. Theoretical calculations indicate that the equilibrium geometry of the ground and excited states of this molecule differ significantly, which plays a decisive role in achieving photo-controlled molecular aggregation. In the ground state, torsion 1 = 120°, whereas torsion 1 = 90° in the excited state. During the transition from ground to excited states, the conformation of compound **1** changes significantly, and the structure of the excited state is significantly more regular than that of the ground state. As a result, the π···π interactions among the excited state molecules tend to be strengthened, leading to the molecules being parallel to each other and forming ordered stacking. Another key factor driving molecular aggregation is the transition of molecular hydrophilicity and hydrophobicity. The space between the molecular side chains in the ground state structure is calculated as 5.0 Å and is more compatible with H_2_O molecules (4.0 Å, Figure 7b). In this case, compound **1** behaves as hydrophilic molecules. The molecules in the ground state can be well dispersed in water, with a particle size not exceeding 10 nm. However, the space becomes smaller and is calculated as 4.1 Å in the excited state, which cannot be well matched with H_2_O molecules. In this case, compound **1** behaves as hydrophobic molecules. Based on these factors, compound **1** can form aggregates through photo irradiation. As the irradiation time increases, aggregates with a 200-nm diameter are formed (Figure 7c). These aggregates can disperse into aqueous solutions again when photo irradiation is removed because compound **1** recovers to be hydrophilic. The entire process of photo-controlled aggregation is a photophysical process based on the transformation of excited state conformation, which provides a reference for controlling excited state conformation to realize photo-controlled molecular self-assembly.

To further study the photo-controlled molecular aggregation caused by the change in excited state conformation in organic solvents, an oil-persulfurated arene molecule (compound **2**, Figure 7d) was used in the research [94]. The theoretical calculation results show that there are obvious changes between ground and excited state conformations. Torsion 1 can change from 118° to 80°, whereas torsion 2 can change from 44° to 15°, proving that these molecules have significant spatial conformational conversion capabilities. The transmission electron microscopy images show that the particle size gradually changes from a few nanometers (10 nm) to hundreds of nanometers (120 nm), which is consistent with the dynamic light scattering results (Figure 7d). Therefore, this type of molecule is proven to have the ability of obvious spatial conformation transformation, which opens the possibility of photo-regulating molecular assemblies. By utilizing this feature, not only can the reversible aggregation of light-driven molecules be achieved, but also photo-controlled crystal growth and realignment can be achieved [95,96].

#### 3.2.2. Photo-Controlled NP Aggregation

**Figure 8:**
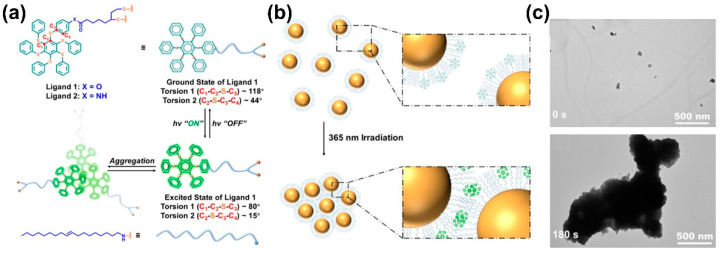
(**a**) Chemical structure of ligand 1 and 2, and the calculated conformational change upon photoexcitation. (**b**) Schematic illustration of photo-induced self-aggregation of 1 Au NPs. (**c**) TEM images of 1 Au NPs under 365 nm light irradiation for 3 min [97].

Inspired by the unique photoexcited, controlled aggregation properties of persulfurated benzenes, if such molecules are used to modify NPs, excited state-controlled self-assembly may be realized [97]. Zhu et al. prepared Au NPs with ligand 1 to modify the surface (Figure 8a). Through the theoretical calculation results, after the conformation change from the ground state to the excited state, torsion 1 (C1-C2-S-C3) can change from 118° to 80°, whereas torsion 2 (C2-S-C3-C4) can change from 44° to 15°. The excited state conformation promotes molecular aggregation of NPs as an assembly driving force. The initial Au NPs can disperse well in toluene solution and form particles with a diameter not greater than 10 nm, whereas these Au NPs particles can self-assemble into large size NP aggregates with a 1 µm diameter after photo irradiation for 3 min (Figure 8b,c). This study proves that molecular aggregation caused by the excited state conformational change can be used as a driving force to drive the self-assembly of NPs, further enriching the molecular assembly system controlled by photophysical processes.

## 4. Summary and Outlook

In this review, we categorized studies on photo-controlled molecular self-assembly into two distinct approaches: (1) photo-controlled self-assembly based on photochemical processes, mainly realized through molecular structure changes to photoswitchable molecules; (2) photo-controlled self-assembly based on photophysical processes, mainly realized through excited state conformational changes to non-traditional photoresponsive molecules. The corresponding advantages and disadvantages of photo-controlled self-assemblies based on these two processes were introduced. Based on these two different photo-controlled self-assembly processes, the self-assembly of photo-controlled NPs was introduced in detail.

Photochemical processes based on traditional photoresponsive molecules were first introduced according to the type of photoreaction. Therefore, three types of photochromic molecules, namely azobenzene, spiropyran, and DAE, typically used to modify NPs to achieve photo-controlled self-assembly of NPs, were discussed. Through covalent bonds or supramolecular interactions, the changes in the structure of photoswitches can further induce the self-assembly of NPs. The reversibility of photoswitch changes can endow the self-assembly of NPs with reversibility. However, several issues still need to be addressed urgently for photochemical processes: (1) self-assembly systems based on photochemical processes mostly rely on UV irradiation. Self-assembly systems controlled by visible light, infrared light, and near-infrared light need to be developed urgently. (2) Effectively avoiding side reactions in photochemical reactions to improve the reversibility and cycles of the self-assembly process is a key factor restricting photo-controlled self-assembly. (3) Current research mainly focuses on photo-induced self-assembly of NPs in solution, and achieving photo-controlled self-assembly in other environments, such as solid and colloidal states, has important research value in in situ and precise regulation.

Meanwhile, self-assembly controlled using photophysical processes is realized by the transformation of excited state conformation; thus, it does not need the introduction of traditional photoresponsive molecules. Polyphenylthiobenzene derivatives were introduced as a kind of molecule with obvious excited state conformational change. Photo-controlled molecular aggregation and photo-controlled NP aggregation based on excited state conformation were summarized. Compared with photochemical processes, research on photophysical processes is nascent; therefore, formidable challenges still exist and continuous efforts are still required. For example, first, developing self-assembled molecular systems driven by molecular spatial conformations is needed. It is crucial to explore the self-assembly of driving molecules based on spatial conformational transformation to achieve the regulation of material structures and function, and meet the growing demand for materials. Second, further understanding of the switch mechanism of molecular spatial conformation in the excited state is required to realize the controllable transformation of spatial conformations. This is of great significance for achieving controllable self-assembled structures, wavelength-dependent responsiveness, and functional design.

In summary, during the past few decades, photo-controlled self-assembly has demonstrated obvious advantages in achieving self-assembly of NPs. Self-assembly controlled using both photochemical and photophysical processes shows great potential in realizing in situ and reversible self-assembly of NPs. In addition to photo-controlled NP self-assembly, further application of photo-controlled technology in fields such as chiral self-assembly, crystal growth, and biomacromolecule self-assembly will have additional research significance. Photo-controlled self-assembly has always been and will continue to be a hot topic in the fields of supramolecular chemistry, nanoscience, and biology, which may lead to the emergence of reconfigurable and programmable materials with more complex structures and functionalities.

## Data Availability

No data was created in this review.

## References

[1] Linko V., Zhang H., Nonappa, Kostiainen M.A., Ikkala O. (2022). From Precision Colloidal Hybrid Materials to Advanced Functional Assemblies. Acc. Chem. Res..

[2] Whitesides G.M., Grzybowski B. (2002). Self-assembly at all scales. Science.

[3] Merindol R., Walther A. (2017). 2 Materials learning from life: Concepts for active, adaptive and autonomous molecular systems. Chem. Soc. Rev..

[4] Montis R., Fusaro L., Falqui A., Hursthouse M.B., Tumanov N., Coles S.J., Threlfall T.L., Horton P.N., Sougrat R., Lafontaine A. (2021). Complex structures arising from the self-assembly of a simple organic salt. Nature.

[5] Boles M.A., Engel M., Talapin D.V. (2016). Self-Assembly of Colloidal Nanocrystals: From Intricate Structures to Functional Materials. Chem. Rev..

[6] Whitesides G.M., Mathias J.P., Seto C.T. (1991). Molecular self-assembly and nanochemistry: A chemical strategy for the synthesis of nanostructures. Science.

[7] Ozin G.A., Hou K., Lotsch B.V., Cademartiri L., Puzzo D.P., Scotognella F., Ghadimi A., Thomson J. (2009). Nanofabrication by self-assembly. Mater. Today.

[8] Lawes P., Boero M., Barhoumi R., Klyatskaya S., Ruben M., Bucher J.P. (2023). Hierarchical Self-Assembly and Conformation of Tb Double-Decker Molecular Magnets: Experiment and Molecular Dynamics. Nanomaterials.

[9] Chen H., Fraser Stoddart J. (2021). From molecular to supramolecular electronics. Nat. Rev. Mater..

[10] Rao A., Roy S., Jain V., Pillai P.P. (2023). Nanoparticle Self-Assembly: From Design Principles to Complex Matter to Functional Materials. ACS Appl. Mater. Interfaces.

[11] Lee M.S., Yee D.W., Ye M., Macfarlane R.J. (2022). Nanoparticle Assembly as a Materials Development Tool. J. Am. Chem. Soc..

[12] Liu W., Semcheddine F., Guo Z., Jiang H., Wang X. (2022). Near-Infrared Light-Triggered Nitric Oxide Nanogenerators for NO-Photothermal Synergistic Cancer Therapy. Nanomaterials.

[13] Weißenfels M., Gemen J., Klajn R. (2021). Dissipative Self-Assembly: Fueling with Chemicals versus Light. Chem.

[14] Ragazzon G., Baroncini M., Silvi S., Venturi M., Credi A. (2015). Light-powered autonomous and directional molecular motion of a dissipative self-assembling system. Nat. Nanotechnol..

[15] Wang Q., Qi Z., Chen M., Qu D.H. (2021). Out-of-equilibrium supramolecular self-assembling systems driven by chemical fuel. Aggregate.

[16] Yagai S., Karatsu T., Kitamura A. (2005). Photocontrollable self-assembly. Chem. Eur. J..

[17] Liang L., Zhao W., Yang X.J., Wu B. (2022). Anion-Coordination-Driven Assembly. Acc. Chem. Res..

[18] Cook T.R., Zheng Y.R., Stang P.J. (2013). Metal-organic frameworks and self-assembled supramolecular coordination complexes: Comparing and contrasting the design, synthesis, and functionality of metal-organic materials. Chem. Rev..

[19] Chen Y., Wang Z., He Y., Yoon Y.J., Jung J., Zhang G., Lin Z. (2018). Light-enabled reversible self-assembly and tunable optical properties of stable hairy nanoparticles. Proc. Natl. Acad. Sci. USA.

[20] Gentili D., Ori G., Ortolani L., Morandi V., Cavallini M. (2017). Cooperative and Reversible Anisotropic Assembly of Gold Nanoparticles by Modulation of Noncovalent Interparticle Interactions. ChemNanoMat.

[21] Montelongo Y., Sikdar D., Ma Y., McIntosh A.J.S., Velleman L., Kucernak A.R., Edel J.B., Kornyshev A.A. (2017). Electrotunable nanoplasmonic liquid mirror. Nat. Mater..

[22] Chen X.M., Feng W.J., Bisoyi H.K., Zhang S., Chen X., Yang H., Li Q. (2022). Light-activated photodeformable supramolecular dissipative self-assemblies. Nat. Commun..

[23] Lubbe A.S., Szymanski W., Feringa B.L. (2017). Recent developments in reversible photoregulation of oligonucleotide structure and function. Chem. Soc. Rev..

[24] Zheng Z.G., Li Y., Bisoyi H.K., Wang L., Bunning T.J., Li Q. (2016). Three-dimensional control of the helical axis of a chiral nematic liquid crystal by light. Nature.

[25] Xu F., Feringa B.L. (2023). Photoresponsive Supramolecular Polymers: From Light-Controlled Small Molecules to Smart Materials. Adv. Mater..

[26] Kundu P.K., Samanta D., Leizrowice R., Margulis B., Zhao H., Borner M., Udayabhaskararao T., Manna D., Klajn R. (2015). Light-controlled self-assembly of non-photoresponsive nanoparticles. Nat. Chem..

[27] Zhao W., Zhang W., Wang R.Y., Ji Y., Wu X., Zhang X. (2019). Photocontrollable Chiral Switching and Selection in Self-Assembled Plasmonic Nanostructure. Adv. Fun. Mater..

[28] Bistervels M.H., Kamp M., Schoenmaker H., Brouwer A.M., Noorduin W.L. (2022). Light-Controlled Nucleation and Shaping of Self-Assembling Nanocomposites. Adv. Mater..

[29] Bansal A., Zhang Y. (2014). Photocontrolled nanoparticle delivery systems for biomedical applications. Acc. Chem. Res..

[30] Yi J., Qin Y., Zhang Y. (2023). Synthesis and Self-Assembly of Hyperbranched Multiarm Copolymer Lysozyme Conjugates Based on Light-Induced Metal-Free Atrp. Nanomaterials.

[31] Jia X., Zhu L. (2023). 9 Photoexcitation-Induced Assembly: A Bottom-Up Physical Strategy for Driving Molecular Motion and Phase Evolution. Acc. Chem. Res..

[32] Bian T., Chu Z., Klajn R. (2020). The Many Ways to Assemble Nanoparticles Using Light. Adv. Mater..

[33] Cheng H.B., Zhang S., Bai E., Cao X., Wang J., Qi J., Liu J., Zhao J., Zhang L., Yoon J. (2022). Future-Oriented Advanced Diarylethene Photoswitches: From Molecular Design to Spontaneous Assembly Systems. Adv. Mater..

[34] Cheng H.B., Zhang S., Qi J., Liang X.J., Yoon J. (2021). Advances in Application of Azobenzene as a Trigger in Biomedicine: Molecular Design and Spontaneous Assembly. Adv. Mater..

[35] Wang L., Li Q. (2018). Photochromism into nanosystems: Towards lighting up the future nanoworld. Chem. Soc. Rev..

[36] Cheng X., Sun R., Yin L., Chai Z., Shi H., Gao M. (2017). Light-Triggered Assembly of Gold Nanoparticles for Photothermal Therapy and Photoacoustic Imaging of Tumors In Vivo. Adv. Mater..

[37] Gentili D., Ori G. (2022). Reversible assembly of nanoparticles: Theory, strategies and computational simulations. Nanoscale.

[38] Wang J., Peled T.S., Klajn R. (2023). Photocleavable Anionic Glues for Light-Responsive Nanoparticle Aggregates. J. Am. Chem. Soc..

[39] Liu M., Yang M., Wan X., Tang Z., Jiang L., Wang S. (2023). From Nanoscopic to Macroscopic Materials by Stimuli-Responsive Nanoparticle Aggregation. Adv. Mater..

[40] Bandara H.M., Burdette S.C. (2012). Photoisomerization in different classes of azobenzene. Chem. Soc. Rev..

[41] Merino E., Ribagorda M. (2012). Control over molecular motion using the cis-trans photoisomerization of the azo group. Beilstein J. Org. Chem..

[42] Stoffelen C., Voskuhl J., Jonkheijm P., Huskens J. (2014). Dual stimuli-responsive self-assembled supramolecular nanoparticles. Angew. Chem. Int. Ed. Engl..

[43] Beharry A.A., Woolley G.A. (2011). Azobenzene photoswitches for biomolecules. Chem. Soc. Rev..

[44] Broichhagen J., Frank J.A., Trauner D. (2015). A roadmap to success in photopharmacology. Acc. Chem. Res..

[45] Klajn R., Bishop K.J., Fialkowski M., Paszewski M., Campbell C.J., Gray T.P., Grzybowski B.A. (2007). Plastic and moldable metals by self-assembly of sticky nanoparticle aggregates. Science.

[46] Klajn R., Bishop K.J., Grzybowski B.A. (2007). Light-controlled self-assembly of reversible and irreversible nanoparticle suprastructures. Proc. Natl. Acad. Sci. USA.

[47] Manna A., Chen P.-L., Akiyama H., Wei T.-X., Tamada K., Knoll W. (2002). Optimized Photoisomerization on Gold Nanoparticles Capped by Unsymmetrical Azobenzene Disulfides. Chem. Mater..

[48] Klajn R., Wesson P.J., Bishop K.J., Grzybowski B.A. (2009). Writing self-erasing images using metastable nanoparticle “inks”. Angew. Chem. Int. Ed. Engl..

[49] Manna D., Udayabhaskararao T., Zhao H., Klajn R. (2015). Orthogonal light-induced self-assembly of nanoparticles using differently substituted azobenzenes. Angew. Chem. Int. Ed. Engl..

[50] Kohntopp A., Dabrowski A., Malicki M., Temps F. (2014). Photoisomerisation and ligand-controlled reversible aggregation of azobenzene-functionalised gold nanoparticles. Chem. Commun..

[51] Zhao H., Sen S., Udayabhaskararao T., Sawczyk M., Kucanda K., Manna D., Kundu P.K., Lee J.W., Kral P., Klajn R. (2016). Reversible trapping and reaction acceleration within dynamically self-assembling nanoflasks. Nat. Nanotechnol..

[52] Zhu L., Yan H., Ang C.Y., Nguyen K.T., Li M., Zhao Y. (2012). Photoswitchable supramolecular catalysis by interparticle host-guest competitive binding. Chem. Eur. J..

[53] Peng L., You M., Wu C., Han D., Ocsoy I., Chen T., Chen Z., Tan W. (2014). Reversible phase transfer of nanoparticles based on photoswitchable host-guest chemistry. ACS Nano.

[54] Park J., Sun L.B., Chen Y.P., Perry Z., Zhou H.C. (2014). Azobenzene-functionalized metal-organic polyhedra for the optically responsive capture and release of guest molecules. Angew. Chem. Int. Ed. Engl..

[55] Suda M., Nakagawa M., Iyoda T., Einaga Y. (2007). Reversible photoswitching of ferromagnetic FePt nanoparticles at room temperature. J. Am. Chem. Soc..

[56] Mogaki R., Okuro K., Aida T. (2017). Adhesive Photoswitch: Selective Photochemical Modulation of Enzymes under Physiological Conditions. J. Am. Chem. Soc..

[57] Liu Q., Zhou Y., Shaukat A., Meng Z., Kyllonen D., Seitz I., Langerreiter D., Kuntze K., Priimagi A., Zheng L. (2023). Optically Controlled Construction of Three-Dimensional Protein Arrays. Angew. Chem. Int. Ed. Engl..

[58] Liu G., Sheng J., Teo W.L., Yang G., Wu H., Li Y., Zhao Y. (2018). Control on Dimensions and Supramolecular Chirality of Self-Assemblies through Light and Metal Ions. J. Am. Chem. Soc..

[59] Yang F., Yue B., Zhu L. (2023). Light-triggered Modulation of Supramolecular Chirality. Chem. Eur. J..

[60] Samanta D., Galaktionova D., Gemen J., Shimon L.J.W., Diskin-Posner Y., Avram L., Kral P., Klajn R. (2018). Reversible chromism of spiropyran in the cavity of a flexible coordination cage. Nat. Commun..

[61] Zhang L., Dai L., Rong Y., Liu Z., Tong D., Huang Y., Chen T. (2015). Light-triggered reversible self-assembly of gold nanoparticle oligomers for tunable SERS. Langmuir.

[62] Hou X.F., Chen X.M., Bisoyi H.K., Qi Q., Xu T., Chen D., Li Q. (2023). Light-Driven Aqueous Dissipative Pseudorotaxanes with Tunable Fluorescence Enabling Deformable Nano-Assemblies. ACS Appl. Mater. Interfaces.

[63] Klajn R. (2014). Spiropyran-based dynamic materials. Chem. Soc. Rev..

[64] Kundu P.K., Das S., Ahrens J., Klajn R. (2016). Controlling the lifetimes of dynamic nanoparticle aggregates by spiropyran functionalization. Nanoscale.

[65] Liu D., Chen W., Sun K., Deng K., Zhang W., Wang Z., Jiang X. (2011). Resettable, multi-readout logic gates based on controllably reversible aggregation of gold nanoparticles. Angew. Chem. Int. Ed. Engl..

[66] Shiraishi Y., Shirakawa E., Tanaka K., Sakamoto H., Ichikawa S., Hirai T. (2014). Spiropyran-modified gold nanoparticles: Reversible size control of aggregates by UV and visible light irradiations. ACS Appl. Mater. Interfaces.

[67] Samanta D., Klajn R. (2016). Aqueous Light-Controlled Self-Assembly of Nanoparticles. Adv. Opt. Mater..

[68] Tian H., Yang S. (2004). Recent progresses on diarylethene based photochromic switches. Chem. Soc. Rev..

[69] Li M., Zhu W.H. (2022). Sterically Hindered Diarylethenes with a Benzobis(thiadiazole) Bridge: Enantiospecific Transformation and Reversible Photosuperstructures. Acc. Chem. Res..

[70] Spangenberg A., Metivier R., Yasukuni R., Shibata K., Brosseau A., Grand J., Aubard J., Yu P., Asahi T., Nakatani K. (2013). Photoswitchable interactions between photochromic organic diarylethene and surface plasmon resonance of gold nanoparticles in hybrid thin films. Phys. Chem. Chem. Phys..

[71] van der Molen S.J., Liao J., Kudernac T., Agustsson J.S., Bernard L., Calame M., van Wees B.J., Feringa B.L., Schonenberger C. (2009). Light-controlled conductance switching of ordered metal-molecule-metal devices. Nano Lett..

[72] Fu H.G., Chen Y., Dai X.Y., Liu Y. (2020). Quaternary Supramolecular Nanoparticles as a Photoerasable Luminescent Ink and Photocontrolled Cell-Imaging Agent. Adv. Opt. Mater..

[73] Liu G., Xu X., Dai X., Jiang C., Zhou Y., Lu L., Liu Y. (2021). Cucurbituril-activated photoreaction of dithienylethene for controllable targeted lysosomal imaging and anti-counterfeiting. Mater. Horiz..

[74] Ferreira P., Ventura B., Barbieri A., Da Silva J.P., Laia C.A.T., Parola A.J., Basilio N. (2019). A Visible-Near-Infrared Light-Responsive Host-Guest Pair with Nanomolar Affinity in Water. Chem. Eur. J..

[75] Wu H., Chen Y., Dai X., Li P., Stoddart J.F., Liu Y. (2019). In Situ Photoconversion of Multicolor Luminescence and Pure White Light Emission Based on Carbon Dot-Supported Supramolecular Assembly. J. Am. Chem. Soc..

[76] Li J.J., Zhang H.Y., Liu G., Dai X., Chen L., Liu Y. (2020). Photocontrolled Light-Harvesting Supramolecular Assembly Based on Aggregation-Induced Excimer Emission. Adv. Opt. Mater..

[77] Yang J., Zhen X., Wang B., Gao X., Ren Z., Wang J., Xie Y., Li J., Peng Q., Pu K. (2018). The influence of the molecular packing on the room temperature phosphorescence of purely organic luminogens. Nat. Commun..

[78] Gu L., Shi H., Gu M., Ling K., Ma H., Cai S., Song L., Ma C., Li H., Xing G. (2018). Dynamic Ultralong Organic Phosphorescence by Photoactivation. Angew. Chem. Int. Ed. Engl..

[79] Xu S., Chen R., Zheng C., Huang W. (2016). Excited State Modulation for Organic Afterglow: Materials and Applications. Adv. Mater..

[80] Hu H., Cheng X., Ma Z., Sijbesma R.P., Ma Z. (2022). Polymer Mechanochromism from Force-Tuned Excited-State Intramolecular Proton Transfer. J. Am. Chem. Soc..

[81] Chen K., Zhang R., Li G., Li B., Ma Y., Sun M., Wang Z., Tang B.Z. (2020). Photo-induced crystallization with emission enhancement (PICEE). Mater. Horiz..

[82] Zhao W., Liu Z., Yu J., Lu X., Lam J.W.Y., Sun J., He Z., Ma H., Tang B.Z. (2021). Turning On Solid-State Luminescence by Phototriggered Subtle Molecular Conformation Variations. Adv. Mater..

[83] Zhang H., Du L., Wang L., Liu J., Wan Q., Kwok R.T.K., Lam J.W.Y., Phillips D.L., Tang B.Z. (2019). Visualization and Manipulation of Molecular Motion in the Solid State through Photoinduced Clusteroluminescence. J. Phys. Chem. Lett..

[84] Jia X., Yue B., Zhou L., Niu X., Wu W., Zhu L. (2020). Fluorescence to multi-colored phosphorescence interconversion of a novel, asterisk-shaped luminogen via multiple external stimuli. Chem. Commun..

[85] Wang J., Zhang M., Han S., Zhu L., Jia X. (2022). Multiple-stimuli-responsive multicolor luminescent self-healing hydrogel and application in information encryption and bioinspired camouflage. J. Mater. Chem. C.

[86] Xu J., Feng H., Teng H., Chen G., Pan S., Chen J., Qian Z. (2018). Reversible Switching between Phosphorescence and Fluorescence in a Unimolecular System Controlled by External Stimuli. Chem. Eur. J..

[87] Wu H., Chi W., Baryshnikov G., Wu B., Gong Y., Zheng D., Li X., Zhao Y., Liu X., Agren H. (2019). Crystal Multi-Conformational Control Through Deformable Carbon-Sulfur Bond for Singlet-Triplet Emissive Tuning. Angew. Chem. Int. Ed. Engl..

[88] Fermi A., Bergamini G., Roy M., Gingras M., Ceroni P. (2014). Turn-on phosphorescence by metal coordination to a multivalent terpyridine ligand: A new paradigm for luminescent sensors. J. Am. Chem. Soc..

[89] Wu H., Zhou Y., Yin L., Hang C., Li X., Agren H., Yi T., Zhang Q., Zhu L. (2017). Helical Self-Assembly-Induced Singlet-Triplet Emissive Switching in a Mechanically Sensitive System. J. Am. Chem. Soc..

[90] Gu J., Yue B., Baryshnikov G.V., Li Z., Zhang M., Shen S., Agren H., Zhu L. (2021). Visualizing Material Processing via Photoexcitation-Controlled Organic-Phase Aggregation-Induced Emission. Research.

[91] Yue B., Jia X., Baryshnikov G.V., Jin X., Feng X., Lu Y., Luo M., Zhang M., Shen S., Agren H. (2022). Photoexcitation-Based Supramolecular Access to Full-Scale Phase-Diagram Structures through in situ Phase-Volume Ratio Phototuning. Angew. Chem. Int. Ed. Engl..

[92] Yue B., Feng X., Wang C., Zhang M., Lin H., Jia X., Zhu L. (2022). In Situ Regulation of Microphase Separation-Recognized Circularly Polarized Luminescence via Photoexcitation-Induced Molecular Aggregation. ACS Nano.

[93] Jia X., Shao C., Bai X., Zhou Q., Wu B., Wang L., Yue B., Zhu H., Zhu L. (2019). Photoexcitation-controlled self-recoverable molecular aggregation for flicker phosphorescence. Proc. Natl. Acad. Sci. USA.

[94] Weng T., Zou Q., Zhang M., Wu B., Baryshnikov G.V., Shen S., Chen X., Agren H., Jia X., Zhu L. (2021). Enhancing the Operability of Photoexcitation-Controlled Aggregation-Induced Emissive Molecules in the Organic Phase. J. Phys. Chem. Lett..

[95] Shen S., Baryshnikov G., Yue B., Wu B., Li X., Zhang M., Ågren H., Zhu L. (2021). Manipulating crystals through photoexcitation-induced molecular realignment. J. Mater. Chem. C.

[96] Shen S., Baryshnikov G.V., Xie Q., Wu B., Lv M., Sun H., Li Z., Agren H., Chen J., Zhu L. (2023). Making multi-twisted luminophores produce persistent room-temperature phosphorescence. Chem. Sci..

[97] Xu X., Zhang M., Li Z., Ye D., Gou L., Zou Q., Zhu L. (2023). Highly efficient light-induced self-assembly of gold nanoparticles promoted by photoexcitation-induced aggregatable ligands. Chem. Commun..

